# Construction of a Support Vector Machine–Based Classifier for Pulmonary Arterial Hypertension Patients

**DOI:** 10.3389/fgene.2021.781011

**Published:** 2021-11-22

**Authors:** Zhenglu Shang, Jiashun Sun, Jingjiao Hui, Yanhua Yu, Xiaoyun Bian, Bowen Yang, Kewu Deng, Li Lin

**Affiliations:** ^1^ Department of Cardiology, Wuxi Huishan District People’s Hospital, Wuxi, China; ^2^ Department of Hospital, Wuxi Huishan District People’s Hospital, Wuxi, China; ^3^ Department of Cardiology, Beijing Tongren Hospital, Beijing, China; ^4^ Department of Cardiology, Shanghai Dongfang Hospital, Shanghai, China

**Keywords:** pulmonary arterial hypertension, SVM-RFE, classifier, biomarker, Na

## Abstract

Pulmonary arterial hypertension (PAH) is a disease leading to right heart failure and death due to increased pulmonary arterial tension and vascular resistance. So far, PAH has not been fully understood, and current treatments are much limited. Gene expression profiles of healthy people and PAH patients in GSE33463 dataset were analyzed in this study. Then 110 differentially expressed genes (DEGs) were obtained. Afterward, the PPI network based on DEGs was constructed, followed by the analysis of functional modules, whose results showed that the genes in the major function modules significantly enriched in immune-related functions. Moreover, four optimal feature genes were screened from the DEGs by support vector machine–recursive feature elimination (SVM-RFE) algorithm (EPB42, IFIT2, FOSB, and SNF1LK). The receiver operating characteristic curve showed that the SVM classifier based on optimal feature genes could effectively distinguish healthy people from PAH patients. Last, the expression of optimal feature genes was analyzed in the GSE33463 dataset and clinical samples. It was found that EPB42 and IFIT2 were highly expressed in PAH patients, while FOSB and SNF1LK were lowly expressed. In conclusion, the four optimal feature genes screened here are potential biomarkers for PAH and are expected to be used in early diagnosis for PAH.

## Introduction

According to the classification of pulmonary hypertension (PH) of the World Health Organization (WHO), pulmonary arterial hypertension (PAH) arising from pulmonary vascular diseases is the first type of PH. The clinical symptoms of PAH mainly include fatigue dyspnea, chest distress, chest pain, syncope, and right heart failure (Galiè et al., 2015, 2016). In accordance with statistics, 11–50 people out of one million suffer from PAH worldwide (Lau et al., 2017). Common PAH types encompass idiopathic PAH (IPAH), heritable PAH (HPAH), drug and toxicant–associated PAH, disease-associated PAH, PAH with long-term calcium channel blocker, pulmonary vein–/blood capillary–involved PAH, and persistent PH of the newborn PAH (Rosenzweig et al., 2019).

Currently, the diagnosis of PAH includes initial screening through Doppler echocardiography, followed by the classification of patients by hemodynamics diagnosis, and etiological diagnosis through ventilation/perfusion scan and nighttime blood saturation determination (Thenappan et al., 2018). Risk stratification should be performed on PAH patients before treatment to evaluate the severe degree. Treatment measures often vary among patients with different types and severe degrees, mainly including general measures (rehabilitation training, vaccination, contraception, etc.), supportive treatment (anticoagulant, diuretic, etc.), and specific therapy targeting four PAH-related molecular pathways (Thenappan et al., 2018; Galiè et al., 2019). However, these treatments can only retard disease progression, instead of completely healing. With advancement in PAH diagnostic technology and treatment methods, patients’ 1- and 3-year survival rates have been remarkably increased (Lau et al., 2017). However, as shown in a survey on PAH patients during 2001–2012 in the United States, despite a decrease in PAH-related hospitalizations, the in-hospital mortality rate remained the same and the treatment expense increased dramatically (Anand et al., 2016). Hence, finding an efficient and economical diagnostic method is helpful to tackle the problems faced currently and to improve people’s understanding of the pathogenesis of PAH.

Because of the gradual mature of sequencing technology, gene sequencing has been widely applied in PAH research. A study analyzed gene expression profiles of pulmonary tissue and found different characteristics in gene expression among pulmonary fibrosis patients with and without PH (Mura et al., 2012). Other than pulmonary tissue, researching gene expression profiles of the PAH patients’ peripheral blood is of great utility. For instance, Hemnes et al. (Hemnes et al., 2015) unearthed mRNAs to distinguish vasodilator-responsive PAH (VR-PAH) and vasodilator–non-responsive PAH (VN-PAH) in the peripheral blood. Construction of a disease classifier based on patients’ gene expression data through the machine learning method has been a hot spot in recent years (Camacho et al., 2018). At present, machine learning has been widely applied in clinical diagnosis of cardiovascular diseases, such as coronary artery calcium scoring (Al’Aref et al., 2019). Integration of key mRNAs and traditional diagnostic methods may increase the accuracy of the latter. In this study, we posited that healthy people and PAH patients possess different characteristics at gene expression level. The dataset of peripheral blood gene expression of healthy people and PAH patients was downloaded from the Gene Expression Omnibus (GEO) database. A support vector machine–recursive feature elimination (SVM-RFE) machine learning algorithm was applied to screen feature genes that could identify healthy people and PAH patients. Afterward, the diagnostic performance of the feature gene-based SVM classifier was analyzed via receiver operating characteristic (ROC) curve. Finally, gene expression was tested in the collected clinical samples. Feature genes in this study can be used for diagnosis and work as potential biomarkers, providing a reference for the subsequent research of PAH mechanism.

## Materials and Methods

### Data Source and Technical Route

**FIGURE 1:**
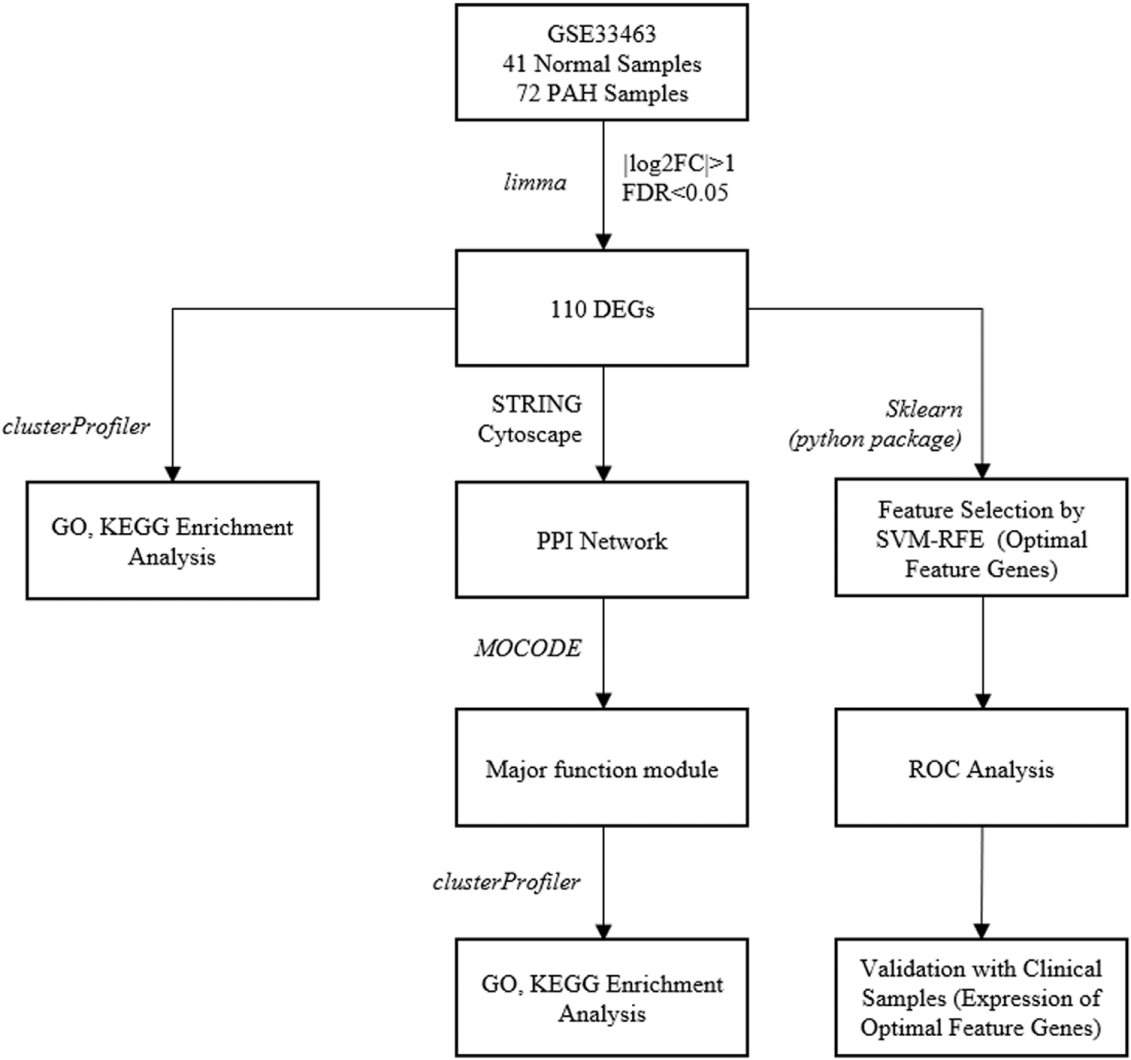
Technical route in this study.

The gene expression data of the GSE33463 dataset were accessed from GEO database (http://www.ncbi.nlm.nih.gov/geo) on 4th April, 2020 (platform No.: GPL6947). The gene expression data of 41 healthy samples and 72 PAH patients were used in the present study. 72 PAH patients included 30 IPAH and 42 systemic sclerosis–associated pulmonary arterial hypertension (SSc-PAH). In the previous context, the technical route in this study is shown in Figure 1.

### Identification of Differentially Expressed Genes

To analyze gene expression changes of PAH patients, differential expression analysis was undertaken on PAH samples with healthy samples as the control. R package Limma was employed (Ritchie et al., 2015), and DEGs were screened with |log2FC| > 1, FDR < 0.05 as threshold values.

### Enrichment Analyses and Construction of Protein–Protein Interaction Network

To explore DEG-involved biological functions, Gene Ontology (GO) and Kyoto Encyclopedia of Genes and Genomes (KEGG) enrichment analyses were performed with R package clusterProfiler (Yu et al., 2012). A *p* value < 0.05 and q value < 0.05 were used to screen significantly enriched items. Meanwhile, the STRING database (version: 11.0) was used to build a PPI network of PAH DEGs (Szklarczyk et al., 2019). The STRING database contains the interaction of known or predicted proteins/genes. The interaction network between the DEGs was predicted with an interaction score >0.4 as the threshold value in this study. The predicted results were visualized through Cytoscape software (Shannon et al., 2003). MOCODE (a plugin in Cytoscape) was applied to screen major functional modules in the PPI network (Chen et al., 2019).

### SVM-RFE Analysis

SVM-RFE is a backward feature elimination method (Guyon et al., 2002; Lin et al., 2017). First, all input features were taken as a feature set F. A classifier model was built based on the SVM algorithm, and the model performance was validated using leave-one-out cross validation (LOOCV). Meanwhile, the weight |*w*| of each feature gene in feature set F was calculated according to the support vector on the SVM classifier hyperplane. The feature gene ranking the last in weight was deleted in the next round of SVM-RFE training, and the remaining feature genes constituted a new feature gene set for re-ranking in the next training. The step was repeated until the feature gene set F was 0. Feature genes were sequenced and selected among PAH DEGs by using the python package *sklearn* (Pedregosa et al., 2012). The key parameters were set as follows: estimator selecting linearSVC, kernel = “linear.” The performance of the PAH classifier was evaluated by four indexes based on the confusion matrix: sensitivity, specificity, accuracy, and MCC.

### Analysis of Classifier Performance and Feature Gene Expression

To validate the diagnostic performance of the optimal feature genes, the ROC curve analysis was performed with R package *time*ROC. First, all healthy samples and the PAH samples were randomly shuffled. Afterward, the predictive efficiency of the single optimal feature gene and SVM model based on the optimal feature gene set was validated by the LOOCV. Finally, the ROC curve was established, and the area under the curve (AUC) was calculated. The AUC value is one of the indexes to assess the predictive performance of the model. Besides, the Wilcoxon test was used to detect the expression differences of optimal feature genes in healthy samples and PAH samples. A *p* value less than 0.05 was considered statistically significant.

### Clinical Sample Collection

This study included 10 PAH patients who received treatment in Wuxi Huishan District People’s Hospital from February 2020 to February 2021. PAH patients met the following criteria: in the resting state, mean pulmonary arterial pressure (mPAP) ≥25 mmHg, pulmonary capillary wedge pressure (PCWP) ≤15 mm Hg, and pulmonary vascular resistance (PVR) ≥3 wood units (McLaughlin et al., 2009). Meanwhile, 10 healthy people without pulmonary disease, autoimmune disease, or other disease history were recruited as healthy control. Samples in this study have been approved by the ethics committee of this hospital. All patients have signed the informed consent.

### Determination of Optimal Feature Gene Expression of Clinical Samples

Peripheral blood mononuclear cells (PBMCs) were isolated from the collected peripheral blood samples through human monocyte separation solution (Axis-Shield, Norway). Following the manufacturer’s instructions, total RNAs of PBMCs were extracted with an RNeasy Mini Kit (Qiagen, German). The concentration of extracted RNA was detected by a NanoDrop One (Thermo Fisher, USA). Afterward, RNA was reverse-transcribed to obtain cDNA with the QuantiTect Reverse Transcription Kit (Qiagen, German) according to the manufacturer’s instructions. Thereafter, two-step RT-qPCR was performed with the QuantiNova SYBR Green PCR Kit (Qiagen, German) to detect the expression of optimal feature genes. Gene primer sequences are listed in Table 1. β-actin was taken as the internal control. The 2^−ΔΔCt^ method was applied to analyze the relative expression of target genes. Three groups of biological replicates were set in each experiment.

**TABLE 1:** Primer sequence of optimal feature genes.

**Primer**	**Forward (5’–3′)**	**Reverse (5’–3′)**
EPB42	CCC​CAT​GGA​TTT​GAA​GTG​CC	AGT​GTG​ACC​AGC​CTT​CCT​AGA
IFIT2	AAG​CAC​CTC​AAA​GGG​CAA​AAC	TCG​GCC​CAT​GTG​ATA​GTA​GAC
FOSB	GTG​AGA​GAT​TTG​CCA​GGG​TC	AGA​GAG​AAG​CCG​TCA​GGT​TG
SNF1LK	GTC​CCT​CGG​AAG​GAA​CTA​GC	CTC​GCG​TTT​TTC​CTT​AGC​TG
β-actin	GTG​GGG​CGC​CCA​GGC​ACC​T	CTT​CCT​TAA​TGT​CAC​GCA​CGA​TTG

### Statistical Analysis

After clinical experimental data were obtained, GraphPad Prism 6.0 was used for analysis. The expression differences of genes in the control group and experimental group were tested by using the *t* test. A *p* value less than 0.05 indicated statistically significant.

## Results

### Identification of DEGs in PAH Patients and Screening of Major Function Modules

**FIGURE 2:**
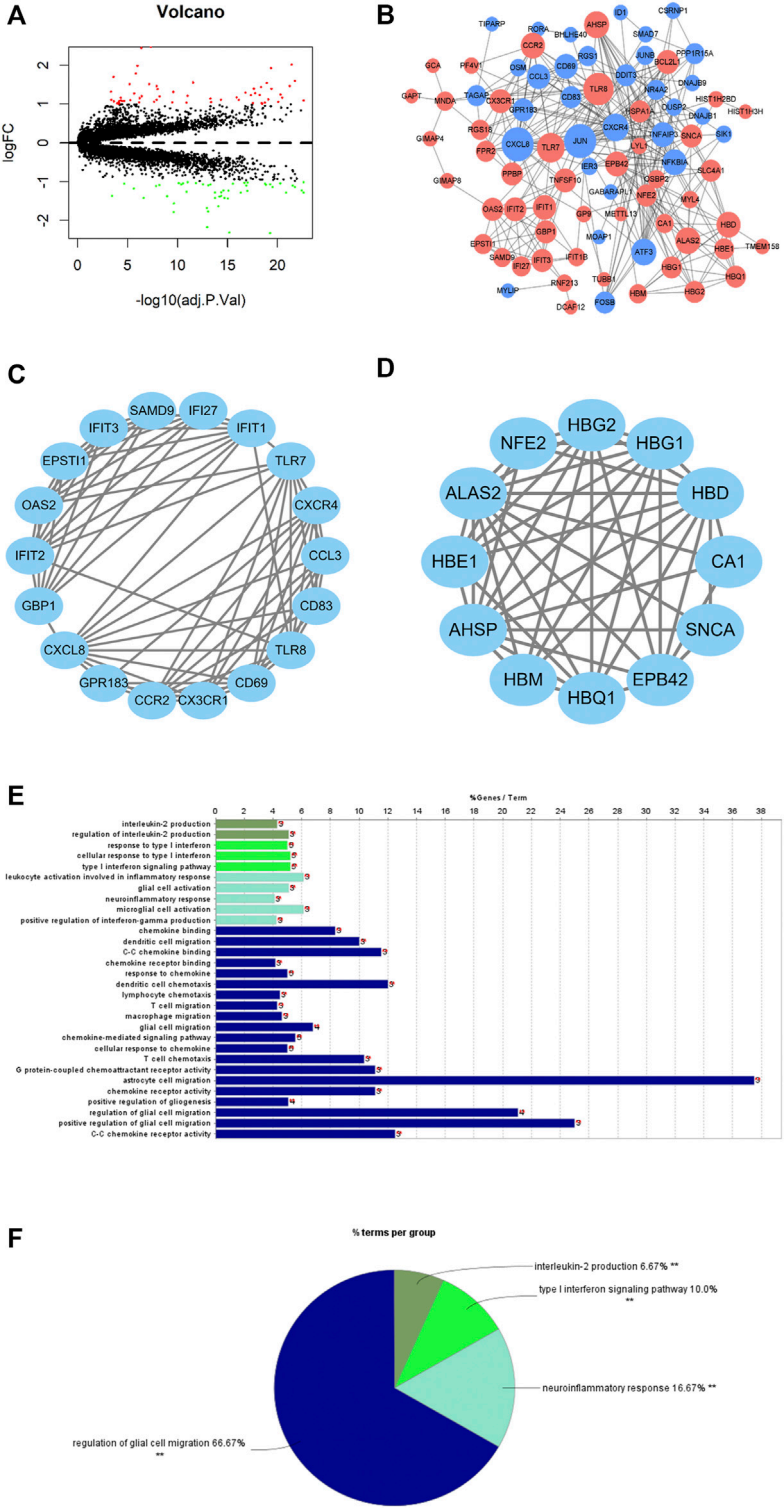
DEGs of PAH patients and DEG functional annotation and enrichment analyses. **(A)** Volcano plot of differential expression analysis of PAH samples relative to healthy samples (red dots: significantly highly expressed genes; green dots: significantly lowly expressed genes). **(B)** PPI network based on PAH DEGs (red nodes: differentially upregulated genes; blue nodes: differentially downregulated genes); node size represents connectivity of this gene in the PPI network. The larger the node, the higher is the connectivity, and the smaller the node, the less is the connectivity. **(C,D)** The major function modules in the PPI network; **(E,F)** GO function enrichment analysis for the genes in the top one major function module.

Differential expression analysis was undertaken on the gene expression profiles of healthy samples and PAH samples. A total of 110 DEGs were obtained (61 upregulated DEGs, 49 downregulated DEGs) (Figure 2A), whose functions were then predicted by the GO and KEGG enrichment analyses (Supplementary Figure S1). A PPI network of DEGs was constructed by using the STRING database (interaction score >0.4). A total of 81 nodes and 300 interacting pairs were obtained (Figure 2B). Then, we used MCODE to screen top two major functional subsets in the PPI network (Figures 2C,D). In top one major functional subset, the TLR7, CXCR4, and CX3CR1 genes were relevant to PAH according to Marasini et al. (2005); Zhang et al. (2020); Zhang et al., (2021). Functional enrichment analysis was undertaken on genes in this subset, and it was found that genes in this subset were mainly enriched in interleukin-2 production, type I interferon signaling pathway, neuroinflammatory response, and the regulation of glial cell migration (Figures 2E,F).

All in all, PAH patients had certain changes in the gene expression level compared with healthy people. The analysis exhibited that the major function modules in the PPI network constructed by DEGs may play a part in immune-related biological functions.

### PAH Feature Genes Screened Using SVM-RFE Analysis

**FIGURE 3:**
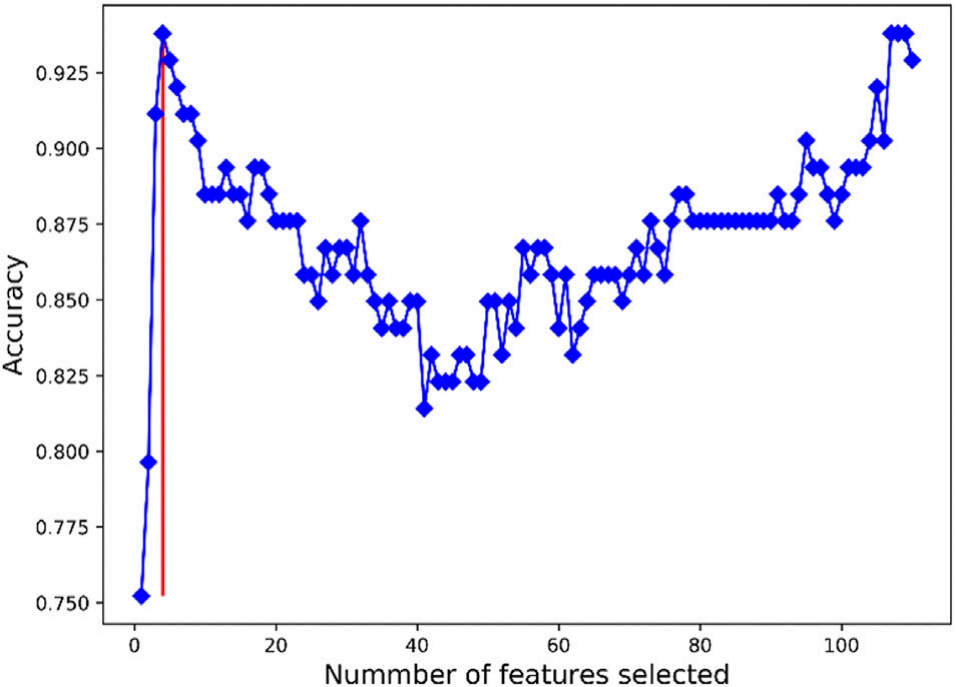
Results of SVM-RFE feature gene selection. *X*-axis refers to the number of feature genes in RFE analysis. *Y*-axis refers to the accuracy of the model. Blue broken line refers to the tendency of accuracy with the number of feature genes. Red vertical line refers to the number of optimal feature genes as the accuracy was the largest.

In a bid to screen feature genes that could be used for the PAH patients’ diagnosis and prognosis prediction, we screened DEGs using SVM-RFE. The accuracy of the classifier reached 0.938 as the number of feature genes = 4, 107, 108, and 109, as shown in Figure 3. The generalization ability of the model declined as the feature number increased. Therefore, four feature genes (EPB42, IFIT2, FOSB, and SNF1LK) were finally selected as the optimal ones. Some data on the four gene-based classifiers are given as follows: sensitivity (0.927), specificity (0.944), accuracy (0.938), and the Matthews correlation coefficient (MCC) value (0.867).

### Analyses of ROC and Optimal Feature Gene Expression

**FIGURE 4:**
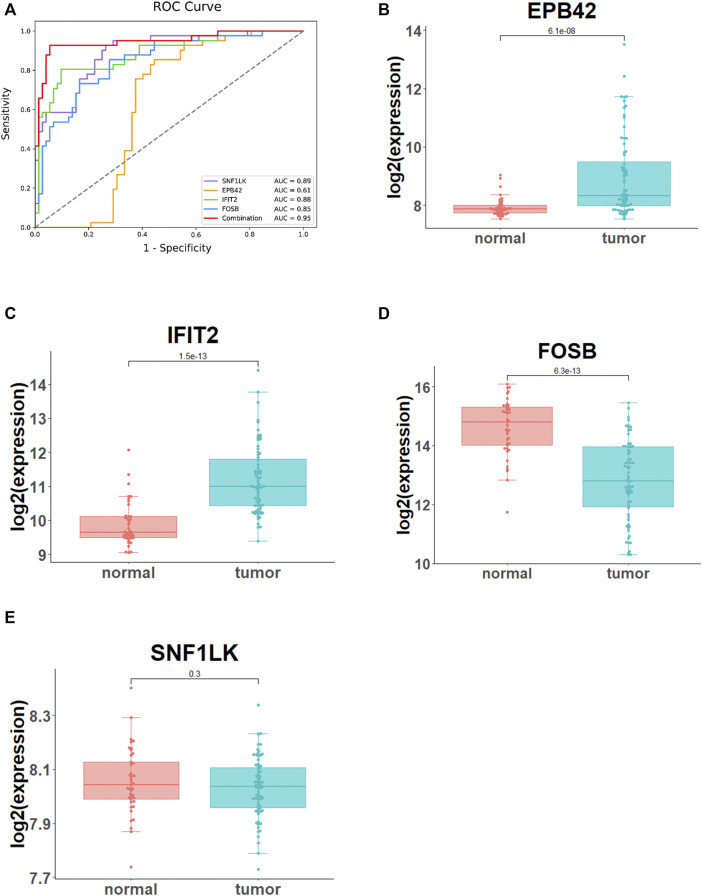
Analyses of ROC and optimal feature gene expression. **(A)** The diagnostic performance of the four optimal feature genes alone and their combination evaluated by ROC analysis. **(B–E)** The expression differences in EPB42, IFIT2, FOSB, and SNF1LK in GSE33463 between normal samples and PAH samples.

For further validation of the diagnostic performance of the four optimal feature genes, here, we compared the predictive effect of four optimal feature genes alone and their combined SVM classifier. ROC analysis showed that the AUC value of four feature gene-based SVM classifiers was 0.95, significantly higher than that of four feature genes alone (Figure 4A). The expression of the four genes was analyzed based on the GSE33463 dataset to probe their expression in PAH patients. As demonstrated by Figures 4B–E, EPB42 and IFIT2 were significantly highly expressed in PAH patients, while FOSB was remarkably lowly expressed. No marked difference was found in SNF1LK expression in healthy people and PAH patients. From the previous results, a combination of the four optimal feature genes dramatically elevated the diagnostic performance of the model. Moreover, EPB42, IFIT2, and FOSB expression levels had marked differences between healthy people and PAH patients.

### Validation of the Expression of Optimal Feature Genes in Clinical Samples

**FIGURE 5:**
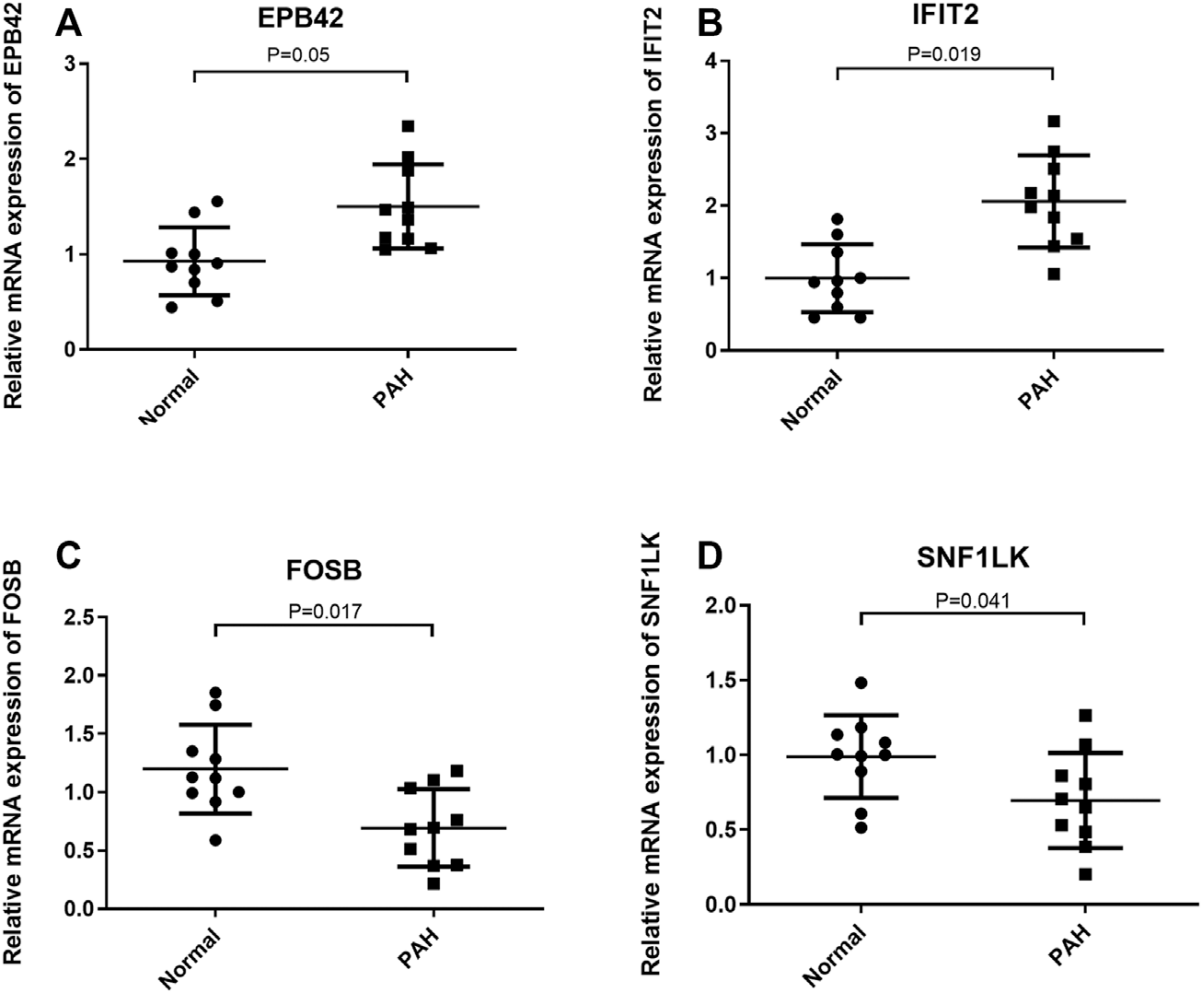
Validation of the expression of optimal feature genes in clinical samples. **(A,B)** Relative to healthy people, EPB42 and IFIT2 are significantly highly expressed in the peripheral blood mononuclear cells of PAH patients. **(C,D)** Relative to healthy people, FOSB and SNF1LK are significantly lowly expressed in the peripheral blood mononuclear cells of PAH patients. *p < 0.05.

The expression of optimal feature genes was further validated in the peripheral blood mononuclear cells of PAH patients by collecting clinical samples. The analysis exhibited that the expression of EPB42 and IFIT2 was significantly upregulated in PAH patients while the expression of FOSB and SNF1LK was markedly downregulated (Figures 5A–D). The results coincided with the analysis results in the GSE33463 dataset.

## Discussion

In recent years, personalized medicine has become increasingly popular for evaluating the patients’ prognosis or therapeutic effect by determining specific disease biomarkers in tissue or blood (Savoia et al., 2017). It is practicable to apply this method for disease diagnosis. For example, four potential diagnostic genes for IPAH were obtained by analyzing the mRNA sequencing data of lung tissue, as evidenced by Zeng et al. (2021). However, the transcriptome analysis of blood samples is more feasible than that of tissue samples in actual clinical diagnosis. Therefore, this work downloaded gene expression profiles of healthy people and PAH patients in the GEO database and established the PAH classifier via a series of bioinformatics analyses.

First, 110 PAH DEGs were obtained by differential expression analysis. The corresponding PPI network analysis revealed a close interplay between these genes. Afterward, function enrichment analysis was performed to analyze the potential functions of these DEGs and the major function modules of the PPI network. The results of the GO enrichment analysis of the top1 function module indicated that several immune-related biological functions were involved in interleukin-2 production, type I interferon signaling pathway, and neuroinflammatory response. Interestingly, dysregulation of cytokines is considered a significant indicator for PAH patients. Likewise, it was reported that many PAH patients suffer from autoimmune and inflammatory diseases (Jafri and Ormiston, 2017; Thenappan et al., 2018), which is consistent with our GO prediction.

After determining the involved biological functions of DEGs in PAH progression, we screened the optimal feature genes to be used for PAH diagnosis through SVM-RFE. SVM-RFE is an algorithm that combines SVM and recursive feature elimination (RFE) proposed by Guyon (Guyon et al., 2002). This algorithm is used for gene selection before classification research. The features are sorted by the SVM classification criteria based on importance or contribution, gradually eliminating the lowest-scored features, iterating repeatedly, and obtaining a subset of features that make the model the most accurate or with the least error (Duan et al., 2005). This method is widely used for the analysis of various disease data (Li et al., 2012; Sahran et al., 2018). Four feature genes were finally acquired: EPB42, IFIT2, FOSB, and SNF1LK. A bioinformatics study presented that IFIT2 is a key gene to SSc-PAH and a potential biomarker, and SSc-PAH is a common PAH relevant to the connective tissue diseases (Zheng et al., 2020). A study illustrated that FOSB shows a highly expressed trend in chronic obstructive pulmonary disease (COPD), while it is lowly expressed in idiopathic pulmonary fibrosis (IPF) (Villaseñor-Altamirano et al., 2020). The FOSB expression varies in different pulmonary diseases and is an underlying biomarker to distinguish COPD-caused PAH and other types of PAH. The other two genes have been rarely researched in PAH. We assessed the expression of optimal feature genes in the GSE33463 dataset and clinical samples. We discovered high expression levels of EPB42 and IFIT2 and low expression levels of FOSB and SNF1LK in PAH patients. According to the above results, four optimal feature genes were taken as PAH classifier and potential PAH biomarkers.

Overall, a four optimal feature gene-based PAH classifier was acquired via a series of bioinformatics analyses based on PAH gene expression data downloaded from the public database. ROC curve analysis suggested that the diagnostic performance of the classifier was favorable and could accurately distinguish healthy people and PAH patients. The expression of these genes was then tested via clinical samples. Few studies have showed concern for the early diagnosis of PAH, while the most common challenge for clinical diagnosis is to determine whether patients had PH or PAH. Right heart catheterization is currently required to accurately diagnose PH and PAH, and the PAH diagnostic–related classifiers built in this study provide a direction for early diagnosis of PAH and reduce patient pain. Clinically, early diagnosis and active intervention can not only slow the progression of PAH but also reduce the fatality rate of disability and may even achieve early cure. However, limitations still exist in this study. For instance, clinical samples were fairly few. Thus, ROC analysis and other subsequent analyses based on these samples are not convincing. In addition, we did not exclude the possibility of other diseases in patients, which may affect the results. We expect to validate the model in more clinical samples and to further explore the feasibility of the model in clinical diagnosis by comparing with the existing methods.

## Data Availability

The original contributions presented in the study are included in the article/Supplementary Material; further inquiries can be directed to the corresponding author.

## Supplementary Material

Supplementary Figure S1 Supplementary Figure of GO and KEGG analyses forof DEGs **(A)** Bubble plot of GO functional annotation of DEGs. The color of circles denotes statistical significance. The size of the circles denotes the number of enriched DEGs. X-axis denotes the percentage of the number of enriched DEGs inout of all genes. BP: biological process. CC: cellular component. MF: molecular function; **(B)** KEGG pathway enrichment analysis of DEGs.
