# Supplementary figures and images for: Construction of a Support Vector Machine–Based Classifier for Pulmonary Arterial Hypertension Patients
Source: Front Genet. 2021 Nov 22;12:781011. doi: 10.3389/fgene.2021.781011 (PMC8647811; doi:10.3389/fgene.2021.781011)

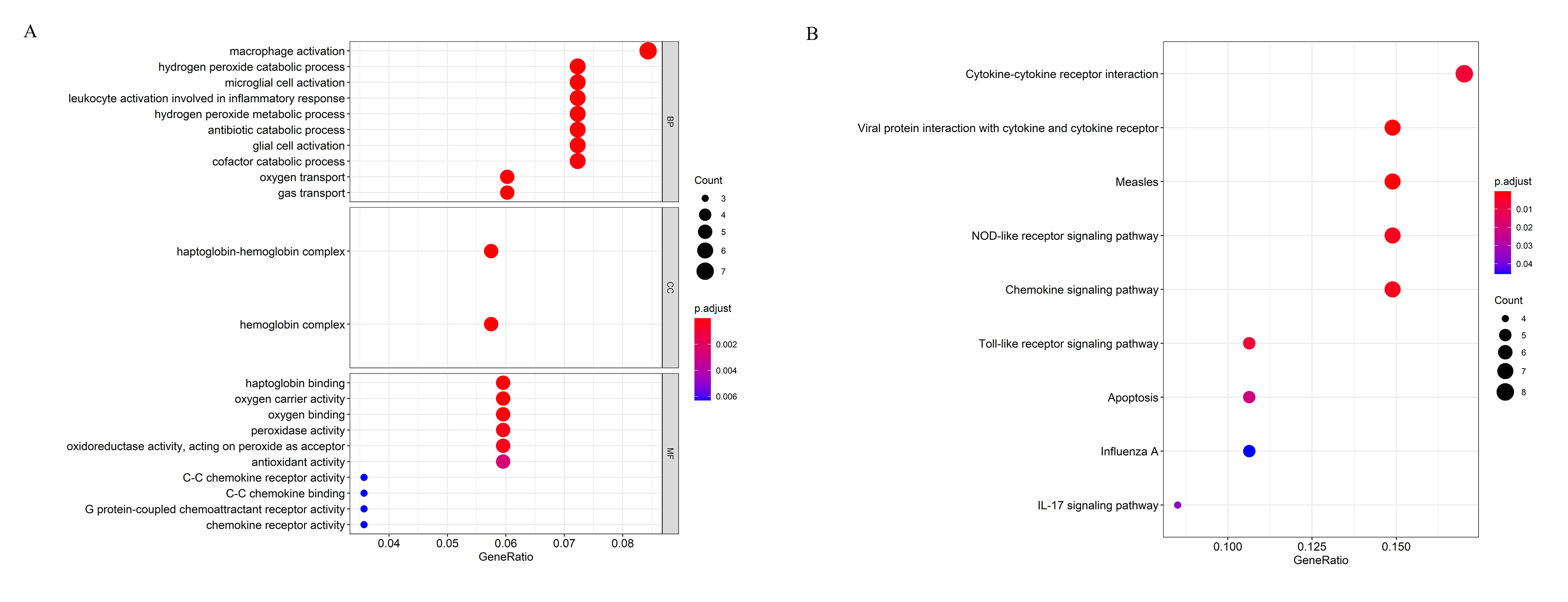

Supplement: Supplementary file 1 [file Image1.tif]
